# Heart Failure Induced by Perinatal Ablation of Cardiac Myosin Light Chain Kinase

**DOI:** 10.3389/fphys.2016.00480

**Published:** 2016-10-26

**Authors:** Yasmin F. K. Islam, Ryan Joseph, Rajib R. Chowdhury, Robert H. Anderson, Hideko Kasahara

**Affiliations:** ^1^Department of Physiology and Functional Genomics, University of FloridaGainesville, FL, USA; ^2^Institute of Genetic Medicine, Newcastle UniversityNewcastle, UK

**Keywords:** heart failure, perinatal, knockout, kinase, Myosin light chain kinase

## Abstract

**Background:** Germline knockout mice are invaluable in understanding the function of the targeted genes. Sometimes, however, unexpected phenotypes are encountered, due in part to the activation of compensatory mechanisms. Germline ablation of cardiac myosin light chain kinase (cMLCK) causes mild cardiac dysfunction with cardiomyocyte hypertrophy, whereas ablation in adult hearts results in acute heart failure with cardiomyocyte atrophy. We hypothesized that compensation after ablation of cMLCK is dependent on developmental staging and perinatal-onset of cMLCK ablation will result in more evident heart failure than germline ablation, but less profound when compared to adult-onset ablation.

**Methods and Results:** The *floxed-Mylk3* gene was ablated at the beginning of the perinatal stage using a single intra-peritoneal tamoxifen injection of 50 mg/kg into pregnant mice on the 19th day of gestation, this being the final day of gestation. The level of cMLCK protein level could no longer be detected 3 days after the injection, with these mice hereafter denoted as the perinatal *Mylk3-KO*. At postnatal day 19, shortly before weaning age, these mice showed reduced cardiac contractility with a fractional shortening 22.8 ± 1.0% (*n* = 7) as opposed to 31.4 ± 1.0% (*n* = 11) in controls. The ratio of the heart weight relative to body weight was significantly increased at 6.68 ± 0.28 mg/g (*n* = 12) relative to the two control groups, 5.90 ± 0.16 (flox/flox, *n* = 11) and 5.81 ± 0.33 (wild/wild/Cre, *n* = 5), accompanied by reduced body weight. Furthermore, their cardiomyocytes were elongated without thickening, with a long-axis of 101.8 ± 2.4 μm (*n* = 320) as opposed to 87.1 ± 1.6 μm (*n* = 360) in the controls.

**Conclusion:** Perinatal ablation of cMLCK produces an increase of heart weight/body weight ratio, a reduction of contractility, and an increase in the expression of fetal genes. The perinatal *Mylk3-K*O cardiomyocytes were elongated in the absence of thickening, differing from the compensatory hypertrophy shown in the germline knockout, and the cardomyocyte thinning shown in adult-inducible knockout.

## Introduction

The incidence of congestive heart failure in childhood ranges from 2.95 to 23.2 in each 1000 patients, of which just over 80% have congenital heart disease, followed by the population with cardiomyopathies (7%) and arrhythmias (2%) (Schmaltz, 2015). The signs and symptoms of heart failure include growth retardation, respiratory distress, and exercise intolerance. The failure in the volume-overloaded heart occurs in the setting of left-to-right shunts, such as ventricular septal defects, persistent patency of the arterial duct, or atrioventricular septal defects. On the other hand, aortic stenosis is the most common cause of pressure-overload heart failure. Complex malformations can induce both volume- and pressure-overloaded heart failure (Hsu and Pearson, 2009a,b; Schmaltz, 2015). As in adult patients, the brain natriuretic peptide (BNP)/N-terminal prohormone BNP is used as a biomarker heart failure in childhood, despite the knowledge that there is an age-dependent variation in normal individuals from ~3000 pg/ml in 0–2 days of age, which drops to ~100 pg/ml between 1 month and 1-year of age (Nir et al., 2009; Schmaltz, 2015).

In clinical settings, a reduction in phosphorylation of myosin light chain 2 (MLC2) has been demonstrated in adult patients with heart failure (Sanbe et al., 1999; Davis et al., 2001; Moss and Fitzsimons, 2006; Stelzer et al., 2006; Scruggs and Solaro, 2011; Sheikh et al., 2012, 2014). It is not yet known, to the best of our knowledge, whether phosphorylation of MLC2, or its responsive kinase, cardiac myosin light chain kinase (cMLCK), is reduced in children with heart failure. When, in genetically modified mice, cMLCK encoded by *Mylk3* gene, was ablated either from the germline or inducibly in adulthood, both populations of cMLCK-deficient mice exhibited heart failure (Warren et al., 2012; Massengill et al., 2016). Knockout in the germline, however, leads to compensatory cardiac hypertrophy, while induction of the knockout in adult mice results in cardiomyocyte atrophy. These studies showed the potential that embryonic hearts have a higher ability to adapt in the absence of cMLCK compared to adult hearts. In this study, we queried if cMLCK ablation during the perinatal stage leads to an intermediate phenotype between those encountered in the setting of embryonic- or adult-onset ablation. We found that perinatal knockout, beginning at embryonic day 19, did indeed produce mice exhibiting moderate heart failure accompanied by elongated cardiomyocytes, but in the absence of any hypertrophy.

## Methods

### Mouse models

A conditional null allele of *Mylk3* was generated by introducing loxP sites spanning exon 5, which was done through homologous recombination in ES cells as described previously (Warren et al., 2012). *Floxed-Mylk3* homozygous mice (*Mylk3*^*flox*∕*flox*^) (Massengill et al., 2016) were bred with transgenic mice carrying the *Cre-ER*™ gene under CMV promoter, *Tg(CAGGS-Cre-ER*™*)* (Hayashi and McMahon, 2002). Subsequent matings between offspring generated *Mylk3*^*flox*∕*flox*^/*Tg-CAGGS-Cre-ER*™, *Mylk3*^*wild*∕*wild*^*/Tg-CAGGS-Cre-ER*™, and *Mylk3*^*flox*∕*flox*^ on a mixed genetic background, mainly C57BL/6J. For perinatal deletion of the *floxed-Mylk3* genes, a single injection of tamoxifen at 50 mg/kg body weight, was administered intra-peritoneally to pregnant mice on the 19th day of gestation, which is the final day of gestation in the mouse. All animal experiments were performed using protocols reviewed and approved by the University of Florida Institutional Animal Care and Use Committee.

### Echocardiogram

Mice were anesthetized with 1.5–2% isoflurane for M-mode ultrasound imaging of the left ventricles using Vevo700 as described previously (Briggs et al., 2008; Takeda et al., 2009; Warren et al., 2012; Massengill et al., 2016).

### Measurements of cardiomyocyte size

Cardiomyocytes were isolated by retrograde perfusion of collagenase as described previously (Takeda et al., 2009; Warren et al., 2012; Massengill et al., 2016). Briefly, the hearts were perfused by Langendorff system with the collagenase digestion buffer (type 2 collagenase 2 mg/ml, in MEM with 10 mM taurine, 3.8 mM creatine, 30 mM 2,3-Butanedione 2-Monoxime, and 20 units of insulin, pH 7.3) at 37⋅C for 30 min. Then the hearts were minced into 8–10 pieces, and further digested with the second collagenase digestion (type 2 collagenase 2 mg/ml, in MEM with 10 mM taurine, 3.8 mM creatine, 30 mM 2,3-Butanedione 2-Monoxime, 20 units of insulin, 0.5% bovine serum albumin, and 0.3 mM CaCl_2_, pH 7.3) at 37⋅C for 10 min. The cells were spun down at 46 × g for 2 min, resuspended in the second digestion buffer without collagenase and loaded onto a 36% Percoll gradient (GE Healthcare). After centrifugation at 290 × g for 10 min, cardiomyocytes were pelleted and non-myocytes were floated on top. The cardiomyocytes were washed twice with the second digestion buffer without collagenase, suspended and plated on the laminin-coated coverglass for 15 min. The cells were immediately fixed with 4% PFA and analyzed for measurement of cell size as described previously (Takeda et al., 2009; Warren et al., 2012; Massengill et al., 2016). All the reagents except for Percoll were obtained from Sigma.

### Western blotting and histological analyses

Western blot analyses and immunostaining were performed with GAPDH (MAB374, EMD Millipore), phospho-MLC2v (gift from Dr. N. Epstein, NIH) (Davis et al., 2001), MLC2 (F109/3E1. ALX-BC-1150-S-L005, Enzo Life Science), and cMLCK (Chan et al., 2008) antibodies. The rabbit affinity-purified cMLCK antibody production has been described previously (Chan et al., 2008). Briefly, the antigen was purified from GST-cMLCK(aa 28-463) following cleavage of GST-tag using thrombin and SDS-PAGE separation followed by electro-elution. Rabbits were injected with the antigen and adjuvants 4 times, and the serum from these animals were affinity-purified using GST-cMLCK(aa 28-463) covalently-coupled beads. 1:4000-diluted antibody was used for Western blotting.

Histological sections (5 μm) were analyzed by hemotoxylin and eosin staining, as well as for fibrosis after Picrosirius red staining, which was performed by heating the tissue sections at 60⋅C for 45 min before deparaffinization and then immersing them in a combination of 0.1% direct red 80 and 0.1% fast green FCF in 1.2% picric acid for 1 h. TUNEL staining was performed using the *In Situ* Cell Death Detection Kit (Roche 11684795910).

### Real-time RT-PCR

Real-time RT-PCR was performed using inventoried Taqman Gene Expression Assays (Thermo Fisher Scientific): Atrial natriuretic factor (ANF) Mm01255748, brain natriuretic peptide (BNP) Mm00435304, and β-myosin heavy chain (βMHC) Mm00600555. All were normalized to β-actin expression (No. 4352933E). Duplicated experiments were averaged.

### Statistical analyses

Data presented are expressed as mean values ± S.E.M. Results were compared using *T*-test or ANOVA with Fisher's *post-hoc* test (SPSS version 24). *p* < 0.05 was considered significant.

## Results

### There is an increase of heart weight relative to the body weight, and overall weight loss in perinatal *Mylk3-KO* mice

**Figure 1:**
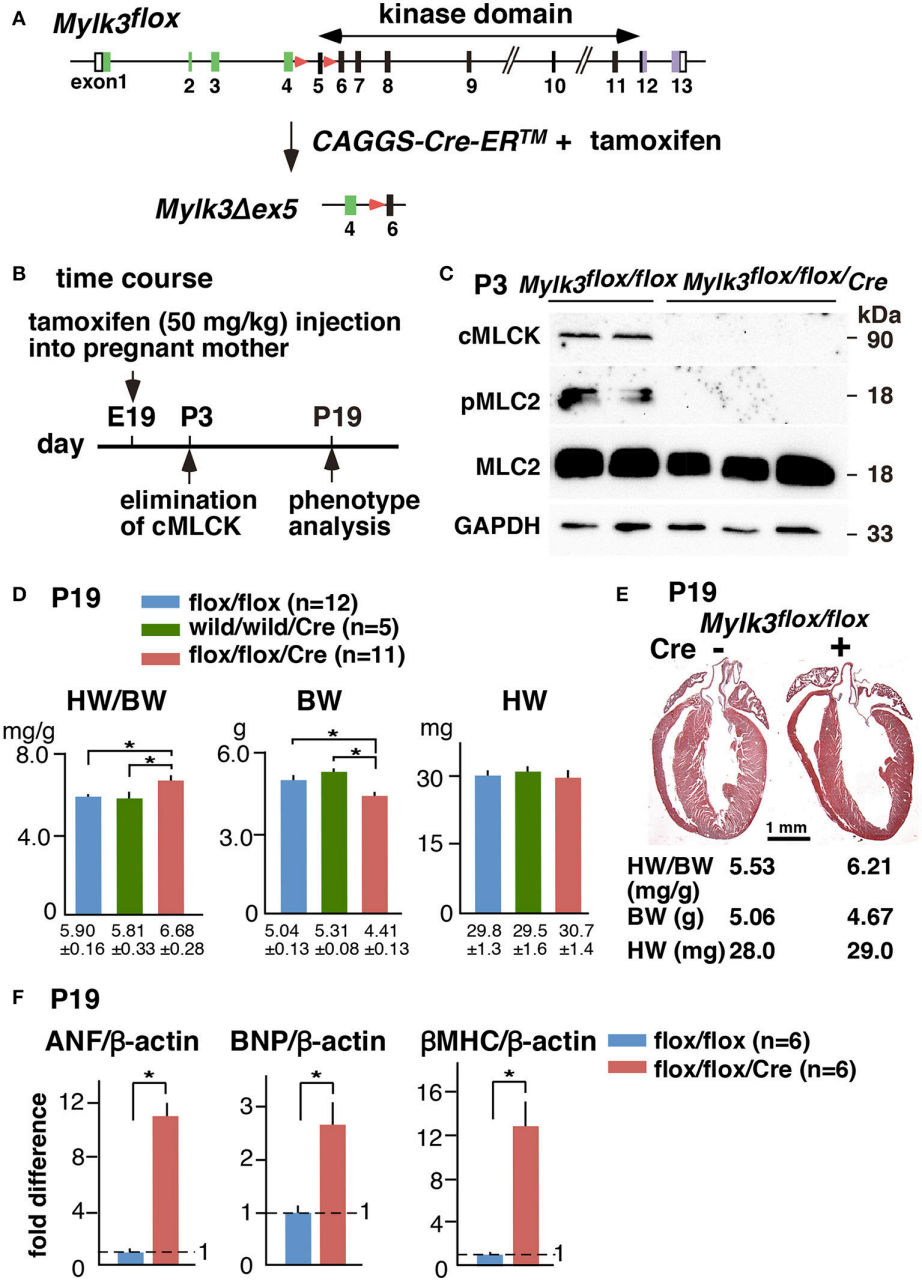
**Experimental design of perinatal inducible *Mylk3-KO* mice leading to acute heart failure. (A)** Strategy for cardiac tamoxifen-inducible perinatal *Mylk3-KO*. **(B)** Timeline of experiments. **(C)** Western blotting demonstrates the expression of cMLCK and MLC2 phosphorylation 3 days after tamoxifen-injection in *Mylk3*^*flox*∕*flox*^ with or without the *CAGGS-Cre-ER*™ transgene. **(D)** Heart weight/body weight (HW/BW) ratio, body weight (BW), and heart weight (HW) of *Mylk3*^*flox*∕*flox*^/*Tg-CAGGS-Cre-ER*™ compared to two controls, *Mylk3*^*flox*∕*flox*^ and *Mylk3*^*wild*∕*wild*^/*Tg-CAGGS-Cre-ER*™. **(E)** Representative sagittal sections of the heart between control (Cre-) and *Mylk3* KO (Cre +) at P19. Bar = 1 mm. **(F)** Real-time RT-PCR shows fold differences in mRNA of ANF, BNP, and β-MHC relative to β-actin with the value in *Mylk3*^*flox*∕*flox*^ defined as 1 (mean ± SE). ^*^p < 0.05 using ANOVA for Panel **(D)** and *T*-test for Panel **(F)**.

To examine the effects of cMLCK in perinatal cardiac function, we generated tamoxifen-inducible *Mylk3* knockout mice that carry homozygous *floxed-Mylk3* alleles and heterozygous Cre-ER™ transgene under the control of the CMV enhancer and the chicken β-globulin promoter (*CAGGS-Cre-ER*™) (Figure 1A). Deletion of floxed-exon 5 resulted in elimination of the first coding exon of the catalytic domain, and a frame-shift of the subsequent downstream exons. In addition, deletion of exon 5 resulted in reduction of cMLCK mRNA (Warren et al., 2012), attributable to a non-sense-mediated mRNA decay (Conti and Izaurralde, 2005), with targeted cMLCK mRNA containing a premature termination codon. Hereafter, conditional deletion of *Mylk3* gene in *Mylk3*^*flox*∕*flox*^*/CAGGS-Cre-ER*™ mice with tamoxifen injection at embryonic day 19, will be described as perinatal *Mylk3-KO*. Of note, this model produces a general knockout of the *Mylk3* gene, although the expression of cMLCK is known to be restricted to the myocardium (Chan et al., 2008).

A single tamoxifen injection into pregnant mice on gestational day 19, at 50 mg/kg, given intraperitoneally, reduced the expression of cMLCK protein and MLC2 phosphorylation by postnatal day 3 (P3), such that it could no longer be detected (Figures 1B,C). During the initial experiment, we noticed that perinatal *Mylk3-KO* mice appeared unhealthy with growth retardation on P19, 2 days before weaning, and there was concern that these mice would die after weaning. Indeed, the ratio of heart weight/body weight (HW/BW) was increased in the perinatal *Mylk3-KO* mice compared to the two control groups, represented by the *Mylk3*^*flox*∕*flox*^ and *Mylk3*^*wild*∕*wild*^*/Tg-CAGGS-Cre-ER*™ mice, mainly due to a decrease in body weight (Figure 1D). The remaining groups of mice, therefore, were analyzed using the same experimental timeline. In Figure 1E, we show representative hearts dissected from the control and perinatal *Mylk3-KO* mice. Expression of fetal genes, often observed in failing hearts, was increased in perinatal *Mylk3-KO* mice, including atrial natriuretic factor (ANF), BNP, and β-myosin heavy chain (βMHC) (Figure 1F). These results indicate that the perinatal *Mylk3-KO* induced heart failure, as shown by the increase of HW/BW ratio, increased fetal gene expression, and the loss of body weight as compared to the controls.

### Contractile dysfunction and cardiomyocyte elongation in the perinatal *Mylk3-KO* mice

**Figure 2:**
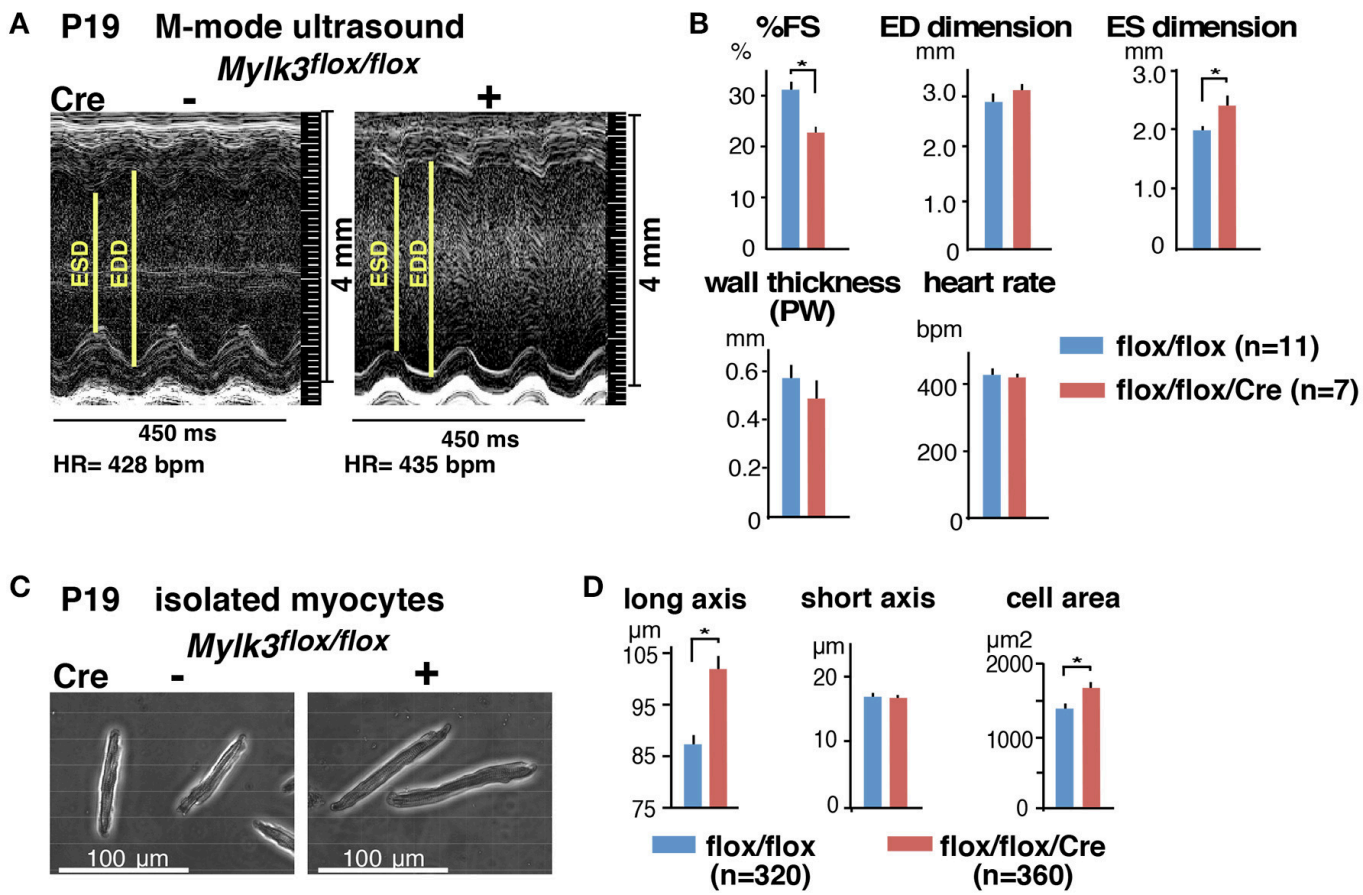
**Reduced contractility and elongation of cardiomyocytes in perinatal *Mylk3-KO* mice. (A)** Representative echocardiographic images, and **(B)** Summary of echocardiographic indices of inducible *Mylk3-KO* mice and control at P19. **(C)** Representative images of isolated cardiomyocytes, and **(D)** summarized data of short- and long axis and cell area size at P19. mean ± SE. ^*^p < 0.05 using *T*-test. FS, fractional shortening; ED, end-diastolic; ES, end-systolic; PW, posterior wall.

**Figure 3:**
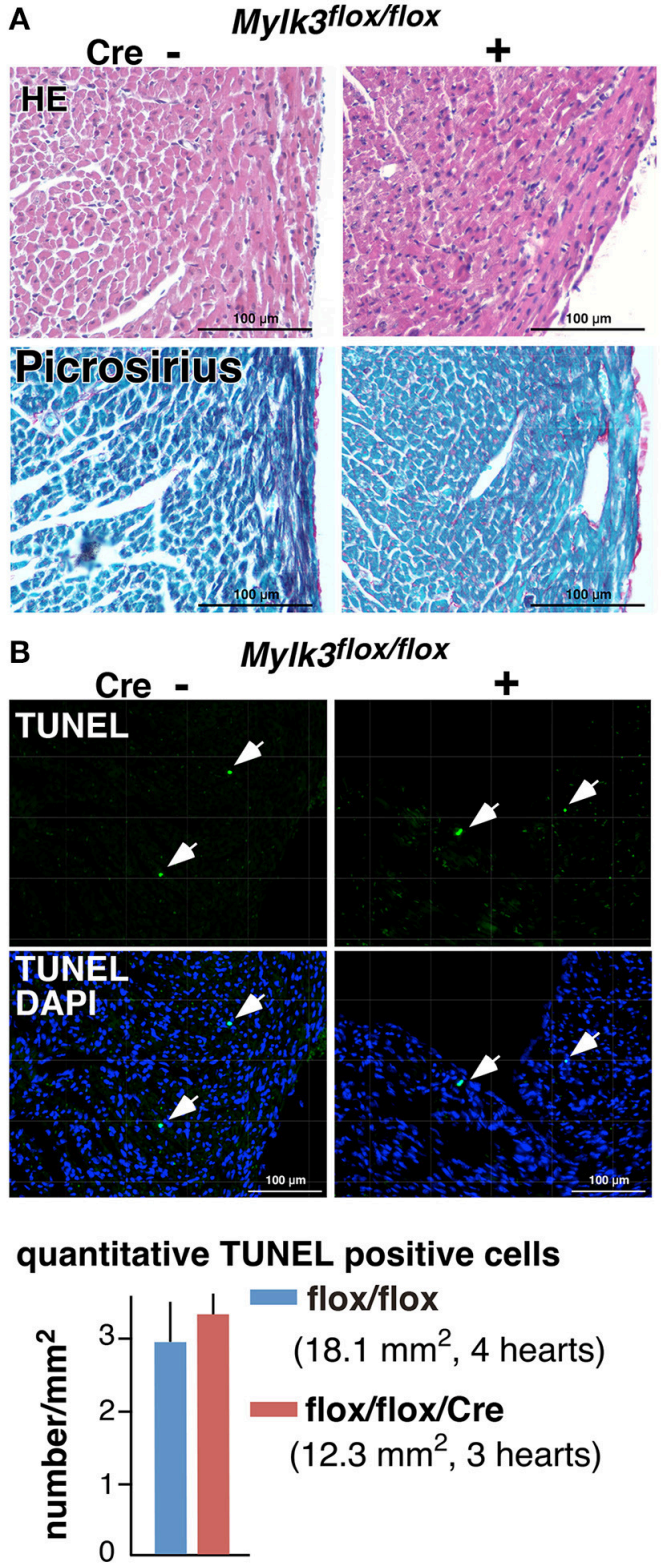
**No apparent interstitial fibrosis or increase of TUNEL-positive cells in perinatal *Mylk3-KO* mice**. **(A)** Representative H&E- and Picrosirius red-stained heart sections from control and perinatal *Mylk3-KO* mice. No apparent fibrosis were seen in inducible marked with arrowheads. **(B)** Representative TUNEL-staining, and the quantitative TUNEL-positive cells indicated by arrows relative to the area size scanned per mouse heart (per mm^2^). Total area size examined, *Mylk3*^*flox*∕*flox*^, 18.1 mm^2^ from 4 mice; *Mylk3*^*flox*∕*flox*^/Cre, 12.3 mm^2^ from 3 mice. Data presented are expressed as mean values ± S.E.

Echocardiography performed on the 19th postnatal day demonstrated a significant reduction in cardiac contractility in perinatal *Mylk3-KO* mice, as measured by %fractional shortening (%FS), and increased systolic dimensions of the left ventricular cavity (Figures 2A,B). Cardiomyocytes isolated from these mice were significantly longer than their controls, albeit without any change in the diameter of the short-axis (Figures 2C,D). Histological analyses, however, failed to show any interstitial fibrosis, nor an increase in TUNEL-positive cells (Figures 3A,B).

### Comparison of the germline, perinatal, and adult-inducible knockout models

Together with our previous studies (Warren et al., 2012; Massengill et al., 2016), we have now analyzed three different *Mylk3* knockout mouse models under similar experimental conditions, with the knockout induced in the germline, in the perinatal period, or at adulthood. The populations show both phenotypic similarities and differences, as we have summarized in Table 1. All three models demonstrate a reduction in cardiac contractility, accompanied with elongation of the cardiomyocytes. On the other hand, the findings regarding the fetal gene expression, interstitial fibrosis, and thickening of the cardiomyocytes were different in the three models. When the knockout was introduced in the germline, it failed to induce expression of fetal genes and interstitial fibrosis, but the cardiomyocytes demonstrated thickening. Knockout in the perinatal period, in contrast, did produce an increase in the expression of fetal genes, but again in the absence of fibrosis, and without producing any change in the short-axis dimensions of cardiomyocytes. Knockout in adult mice produced expression of fetal genes, fibrosis, and cardiomyocyte atrophy. These results showed that, when the *Mylk3* gene is eliminated in early stages of development, it is most effectively compensated. Compensation, in contrast, is ineffective when the *Mylk3* gene is knocked-out in adult mice. Notably, we included two controls in the inducible knockout studies, namely flox/flox and wild/wild/Cre mice with tamoxifen injection in the present and previous studies (Massengill et al., 2016), so as to eliminate any effects of the potential toxicity of tamoxifen and/or Cre transgene for cardiac contractility. Neither of the control populations showed any significant differences in HW/BW ratio or cardiac contractility.

**Table 1:** **Summary of the phenotypes displayed in three *Mylk3* knockout models**.

***Mylk3* knockout types**	**Age analyzed**	**Duration of absence of cMLCK**	**Fetal gene**	**Contractility**	**Fibrosis**	**Long axis**	**Short axis**	**TUNEL**
Germline	3 month	>3 months	No change	Reduced	No	Elongated	Thickened	N/A
Perinatal	P19	16–19 days	Increased	Reduced	No	Elongated	No change	No change
Adult	10–12 weeks	4–5 days or 11–12 days	Increased	Reduced	Yes	Elongated	Thinned	Increased

## Discussion

We have now shown that elimination of cMLCK in mice in the perinatal period results in an increase of heart weight/body weight ratio with decreased body weight, reduction of contractility accompanied by elongated cardiomyocytes, and an increase in the expression of fetal genes. Under sustained pressure overload, adaptive cardiomyocyte hypertrophy transits to maladaptive elongation of cardiomyocytes, and persistent reductions in contractile force. Such cardiac remodeling is thought to involve an initial adaptive addition of sarcomeres in parallel, thus thickening the cardiomyocytes, and a subsequent addition of sarcomere in series to produce elongatation (Diwan and Dorn, 2007; Balasubramanian et al., 2009; Russell et al., 2010; Rosca et al., 2013).

In adult cardiomyocytes, with their semi-crystalline architecture, and under the continuous production of myocardial force, the exact processes of cardiac remodeling remain unknown. In adult mice, nonetheless, expression of cMLCK has been shown to be reduced after pressure overload by ~80% within 1 week. This effect persists, coinciding with the transition from compensated to decompensated hypertrophic heart failure (Warren et al., 2012). Furthermore, our recent study showed that elimination of cMLCK in adult mice with age of 10–12 weeks led to acute heart failure accompanied by cardiomyocyte atrophy with elongated and thinner cardiomyocytes within 1 week after tamoxifen injection. The heart failure persisted with slight further progression in cardiac contractility demonstrated in echocardiogram 2 weeks after tamoxifen injection. Furthermore, adult *Mylk3-KO* mice knockout mice fail to demonstrate adaptive pressure overloaded cardiomyocyte thickening (Massengill et al., 2016).

We could not examine the effects of pressure overload in neonatal hearts, such as seen in aortic stenosis in children, due to technical burdens. To best of our knowledge, it is unknown whether heart failure as seen in childhood also involves reduction of phosphorylation of MLC and cMLCK. We noted, nonetheless, that 19 days after tamoxifen injection into perinatal *Mylk3-KO* mice, there was a heart failure with increase of the heart weight/body weight ratio, reduction of contractility with increase of both systolic and diastolic left ventricular cavity with elongation but without thinning of cardiomyocytes, and increase of fetal gene expression compared to controls. We cannot explain why the heart weight was not increased despite the increase of surface area size and elongation of cardiomyocytes, but it might be possible that individual cardiomyocytes from perinatal *Mylk3-KO* mice are less heavy than those from controls.

In the perinatal *Mylk3-KO* mice, we were unable to detect interstitial fibrosis in the ventricles. We have previously analyzed mice with perinatal knockout of transcription factor, Nkx2-5, using *floxed-Nkx2-5* mice. Nkx2-5 regulates multiple downstream targets including cMLCK (Chan et al., 2008), and perinatal *Nkx2-5-KO* mice demonstrate more profound heart failure compared to *perinatal Mylk3-KO* mice, with a 1.44 fold increase of HW/BW ratio at P12 (Briggs et al., 2008). The perinatal *Nkx2-5* knockout mice did not show any apparent interstitial fibrosis (Briggs et al., 2008). To best of our knowledge, there are no additional reports describing genetically-induced perinatal models of heart failure in mice. Absence of fibrosis, however, has also been observed in neonatal mice following cardiac injury, such as myocardial infarction or partial ventricular resection, partly due to cardiomyocyte regeneration persisting in neonatal hearts within a few days after birth, and difference in extracellular matrix composition and immune responses (Porrello and Olson, 2014).

In mice, cMLCK is expressed from an early embryonic stage, at least beyond E10.5, and is restricted to the hearts when examined in major organs using Northern blotting (Chan et al., 2008). Because of this, we presume that the reduction in body weight observed in the perinatal *Mylk3-KO* mice is due to heart failure, but we cannot rule out the possibility that cMLCK may directly regulate body weight by unknown mechanisms. Additional experiments are now needed further to address and explore the mechanisms underscoring the growth retardation noted in these mice.

To mitigate the possibility that a high dose of tamoxifen, such as 80 mg/kg/day given over 5 days, would result in heart failure (Koitabashi et al., 2009), we utilized a substantially lower dose of tamoxifen, namely 50 mg/kg/day given but once. We also included control wildtype mice with *CAGGS-Cre-ER*™ mice, which showed no change in HW/BW ratio compared to another control, flox/flox mice, after tamoxifen injection, thus eliminating the possibility that the heart failure was due to presence of the *CAGGS-Cre-ER*™ transgene.

We recognize the limitations in our current study. The mechanisms underlying the loss of cMLCK leading to cardiomyocyte elongation are currently unknown, and future experiments are required to elucidate these processes. We have not examined perinatal *Mylk3-KO* beyond 19 days of age because these mice were noted to be unhealthy for weaning on day 21. The remodeling and functional differences between the germline and perinatal *Mylk3-KO* models may be due to the developmental age studied. Further experiments comparing remodeling and cardiac function in these two knockout models at the same ages, therefore, are highly desirable. Based on the profound heart failure seen in adult-inducible *Mylk3-KO* mice, nonetheless, we speculate that their cardiac function is unlikely to improve in later life.

In summary, we have demonstrated that the cMLCK plays essential roles in cardiac function during the perinatal period. Our experiments point to the need for further clinical studies in children with heart failure, exploring the phosphorylation of MLC and reduction of cMLCK.

## Author contributions

Experiments were designed and performed by YI, RJ, RC and HK. The manuscript was prepared by YI, RA, and HK.

## Funding

This work was supported by American Heart Association (14GRNAT20380822 to HK), University of Florida (T35 HL007489 to YI, and Opportunity Fund to HK).

### Conflict of interest statement

The authors declare that the research was conducted in the absence of any commercial or financial relationships that could be construed as a potential conflict of interest.
